# Alpha-Particle Emitting ^213^Bi-Anti-EGFR Immunoconjugates Eradicate Tumor Cells Independent of Oxygenation

**DOI:** 10.1371/journal.pone.0064730

**Published:** 2013-05-28

**Authors:** Christian Wulbrand, Christof Seidl, Florian C. Gaertner, Frank Bruchertseifer, Alfred Morgenstern, Markus Essler, Reingard Senekowitsch-Schmidtke

**Affiliations:** 1 Department of Nuclear Medicine, Technische Universität München, Munich, Germany; 2 European Commission, Joint Research Centre, Institute for Transuranium Elements, Karlsruhe, Germany; Technische Universitaet Muenchen, Germany

## Abstract

Hypoxia is a central problem in tumor treatment because hypoxic cells are less sensitive to chemo- and radiotherapy than normoxic cells. Radioresistance of hypoxic tumor cells is due to reduced sensitivity towards low Linear Energy Transfer (LET) radiation. High LET α-emitters are thought to eradicate tumor cells independent of cellular oxygenation. Therefore, the aim of this study was to demonstrate that cell-bound α-particle emitting ^213^Bi immunoconjugates kill hypoxic and normoxic CAL33 tumor cells with identical efficiency. For that purpose CAL33 cells were incubated with ^213^Bi-anti-EGFR-MAb or irradiated with photons with a nominal energy of 6 MeV both under hypoxic and normoxic conditions. Oxygenation of cells was checked via the hypoxia-associated marker HIF-1α. Survival of cells was analysed using the clonogenic assay. Cell viability was monitored with the WST colorimetric assay. Results were evaluated statistically using a t-test and a Generalized Linear Mixed Model (GLMM). Survival and viability of CAL33 cells decreased both after incubation with increasing ^213^Bi-anti-EGFR-MAb activity concentrations (9.25 kBq/ml–1.48 MBq/ml) and irradiation with increasing doses of photons (0.5–12 Gy). Following photon irradiation survival and viability of normoxic cells were significantly lower than those of hypoxic cells at all doses analysed. In contrast, cell death induced by ^213^Bi-anti-EGFR-MAb turned out to be independent of cellular oxygenation. These results demonstrate that α-particle emitting ^213^Bi-immunoconjugates eradicate hypoxic tumor cells as effective as normoxic cells. Therefore, ^213^Bi-radioimmunotherapy seems to be an appropriate strategy for treatment of hypoxic tumors.

## Introduction

In solid tumors hypoxia results from accelerated proliferation combined with high metabolic activities and poor oxygenation due to insufficient blood supply [1], [2]. In normoxic tissues the mean partial pressure of oxygen (p[O_2_]) is roughly 40 mmHg, while the p[O_2_] in hypoxic tumor areas is below <10 mmHg [3], [4]. Hypoxic cells within a tumor are resistant to radiotherapy, thus negatively influencing the therapeutic outcome [3]. Radioresistance is supposed to appear at p[O_2_] <10 mmHg [4], [5]. It can be quantified by the oxygen enhancement ratio (OER) expressing the ratio of radiation dose required under hypoxia and normoxia to produce the same biological effect [6]. On the one hand, lower sensitivity towards ionizing radiation is explained by the oxygen effect [7]. In cells lacking oxygen DNA damage is less severe because of (i) lower levels of radicals produced by ionizing radiation that cause indirect DNA strand breaks and (ii) absent fixation of DNA damage by oxygen [1]. On the other hand, hypoxia-related tumor radioresistance is triggered by biological signaling pathways. The hypoxia-inducible transcription factor HIF-1 modulates more than 100 genes that play a crucial role in adaption to hypoxia [7], [8]. Moreover, HIF-1 becomes upregulated after radiation therapy of tumors. HIF-1 induces cytokines, which are involved in protection of endothelial cells from the effects of radiation [9]. Altogether, HIF-1 activation leads to an increased resistance to radio- and chemotherapy, increased local aggressive growth and an increased risk of metastatic disease [7], [8].

Previous approaches to overcome radioresistance were aimed at reducing hypoxia. However, hyperbaric oxygen, red blood cell transfusion, erythropoiesis-stimulating factors as well as inhalation of hyperoxic gases with vasodilating drugs did not turn out satisfactory in clinical settings [10].

Therefore, in recent approaches molecular processes that trigger radioresistance of hypoxic tumors are exploited in terms of development of strategies to overcome radioresistance [1]. This includes compounds that inhibit HIF-1 activity through diverse molecular mechanisms. For example, the inhibitor of HSP-1 synthesis and stability YC-1 can help to overcome radioresistance of hypoxic tumour cells [11]. Besides, radiosensitizers like nitroimidazole derivatives as well as C-1027 and KNK437 have revealed promising results in terms of enhancement of cytotoxic effects of ionizing radiation under hypoxia [1], [12], [13], [14]. The hypoxic cytotoxin tirapazamine showed benefits in patients with head and neck cancer [15]. Also suicide gene therapy with the bacterial cytosine deaminase/5-fluorocytosine gene therapy system under the control of a hypoxia-responsive promoter significantly enhanced the therapeutic effects of radiotherapy [16].

Another therapeutic strategy involves fractionated irradiation of hypoxic tumors. As a consequence of radiotherapy tumors become reoxygenated [9]. Accordingly fractionated irradiation of tumors was demonstrated to decrease hypoxia [17].

Irradiation of hypoxic tumors with high Linear Energy Transfer (LET) radiation is an exciting therapeutic option. Because OER decreases with increasing LET [18] high LET Auger electrons or α-particles are thought to directly damage DNA and thus to eradicate tumor cells independent of cellular oxygenation. As shown recently, hypoxic MCF-7 tumor cells are damaged selectively and severely by the hypoxia tracer ^64^Cu-diacetyl-bis(N(4)-methylthiosemicarbazone) (^64^Cu-ATSM) due to emission of Auger electrons [19].

Nevertheless, among high LET-emitters α-particle emitters are the most promising ones in terms of eradication of tumor cells independent of cellular oxygenation. Efficacy of targeted tumor therapy with α-emitters such as ^225^Ac, ^213^Bi, ^212^Bi/^212^Pb, ^211^At or ^227^Th was demonstrated in an increasing number of experimental and clinical studies [20]. Clinical trials using α-emitter antibody or peptide conjugates have been conducted in the treatment of melanoma [21], gliomas [22], [23], acute myeloid leukaemia [24] and ovarian carcinoma [25].

In a multitude of tumor types, such as head and neck squamous cell carcinoma (HNSCC) or pancreatic cancer, hypoxia impedes efficiency of conventional radiation therapy [3]. Because HNSCC cells are characterized by overexpression of EGFR [26], therapy with α-emitter immunoconjugates targeting EGFR is a promising concept. We have compared efficacy of photon irradiation vs. treatment with ^213^Bi-anti-EGFR immunoconjugates in CAL33 HNSCC cells under normoxic and hypoxic conditions in order to prove oxygen-independent cytotoxicity of the α-emitting ^213^Bi-anti-EGFR-MAb.

The results of this study indeed show for the first time that internal irradiation of CAL33 tumor cells with alpha-emitter ^213^Bi-anti-EGFR immunoconjugates is equally efficient in eradication of hypoxic and normoxic cells. Previous work on that subject that has been carried out some 20 to 40 years ago exclusively used external sources of irradiation implicating methodical inadequateness. Therefore ^213^Bi-radioimmunotherapy could be a promising future option for treatment of hypoxic tumors.

## Materials and Methods

### Cell culture

The human head and neck squamous cell carcinoma (HNSCC) cell line CAL33 was obtained from the Leibniz Institute DSMZ (German collection of microorganisms and cell cultures). CAL33 cells were shown to over-express EGFR. The cells were grown in RPMI culture medium (Biochrom, Germany) supplemented with 10% FCS (Biochrom, Germany) and 5% non-essential amino acids (NEA; Biochrom, Germany) at 37°C in a humidified atmosphere with 95% air and 5% CO_2_. These conditions are referred to as normoxic.

### Cultivation of cells under hypoxic conditions

**Figure 1:**
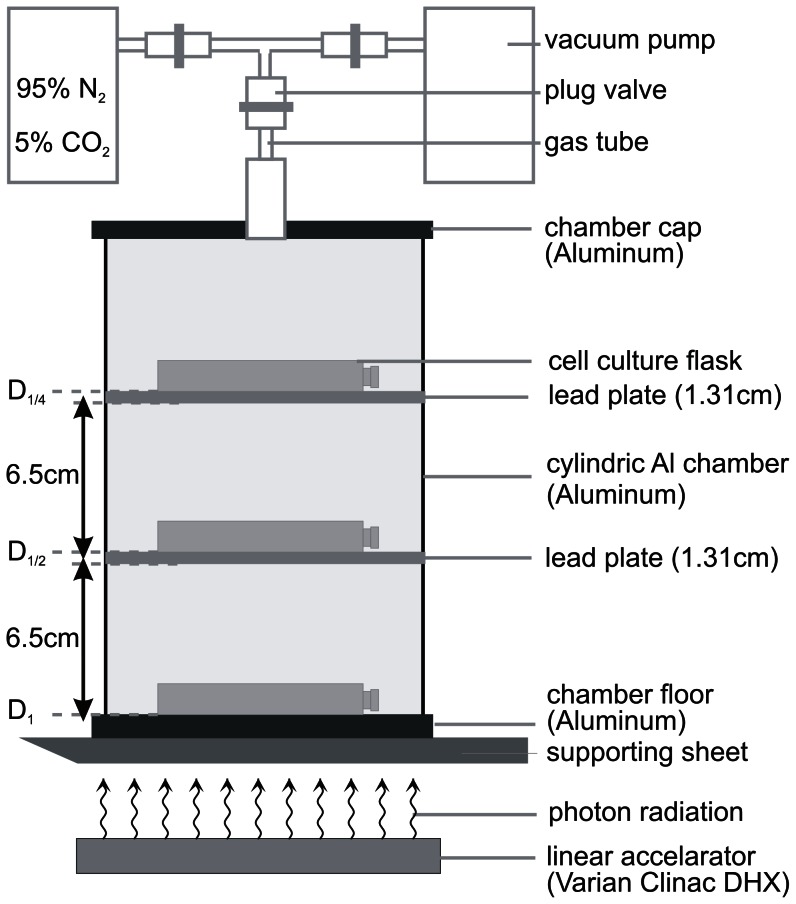
Photon irradiation of CAL33 cells in the aluminium hypoxic chamber. Culture flasks were arranged in three levels, separated by lead plates (thickness 1.31 cm; distance 6.5 cm). Thus, irradiation doses were reduced by half (D_1_, D_1/2_, D_1/4_), respectively, as confirmed by dosimetric measurements. Cells were irradiated with three different doses in one irradiation experiment. Hypoxia was established by repeated aspiration of air and influx of 95% N_2_, 5% CO_2_.

CAL33 cells were seeded in culture flasks or multi-well plates and allowed to adhere overnight under normoxic conditions. For adjustment of hypoxia, cells were placed into an air-tight aluminium chamber at 37°C. From this chamber the air was evacuated with a vacuum pump and replaced with a mixture of gas consisting of 95% N_2_ and 5% CO_2_ (Fig. 1). After a total of 11 cycles (duration 22 min) of air evacuation and gas-influx the oxygen concentration in the culture medium was less than 0.66%/5 mmHg. An oxygen concentration of 1%/10 mmHg was already reached after 9 min of air evacuation and gas-influx [27], [D. Schilling, personal communication]. The beginning of air evacuation was referred to time after incubation under hypoxic conditions.

### Detection of the hypoxia marker HIF-1α in CAL33 cells

HIF-1α, found in mammalian cells cultured under reduced p[O_2_], is part of the heterodimeric HIF-1 complex. Under normoxic conditions HIF-1α is rapidly degraded by the ubiquitin-proteasome system [11].

Intensity of HIF-1α expression was checked via Western blot analysis to indicate the hypoxic status of the CAL33 cells. The HIF-1α signal was analysed immediately after incubation of CAL33 cells for different times (0–24 h) under hypoxic conditions. Because Co^2+^ induces HIF-1α expression in normoxic cells [28], CAL33 cells treated with CoCl_2_ (200 µM, 5 h) were used as positive control. To determine the kinetics of HIF-1α degradation in CAL33 cells that were incubated under hypoxic conditions for 3 h, the HIF-1α signal was analysed at different times (0–180 min) after release from hypoxia and incubation under normoxic conditions.

**Figure 2:**
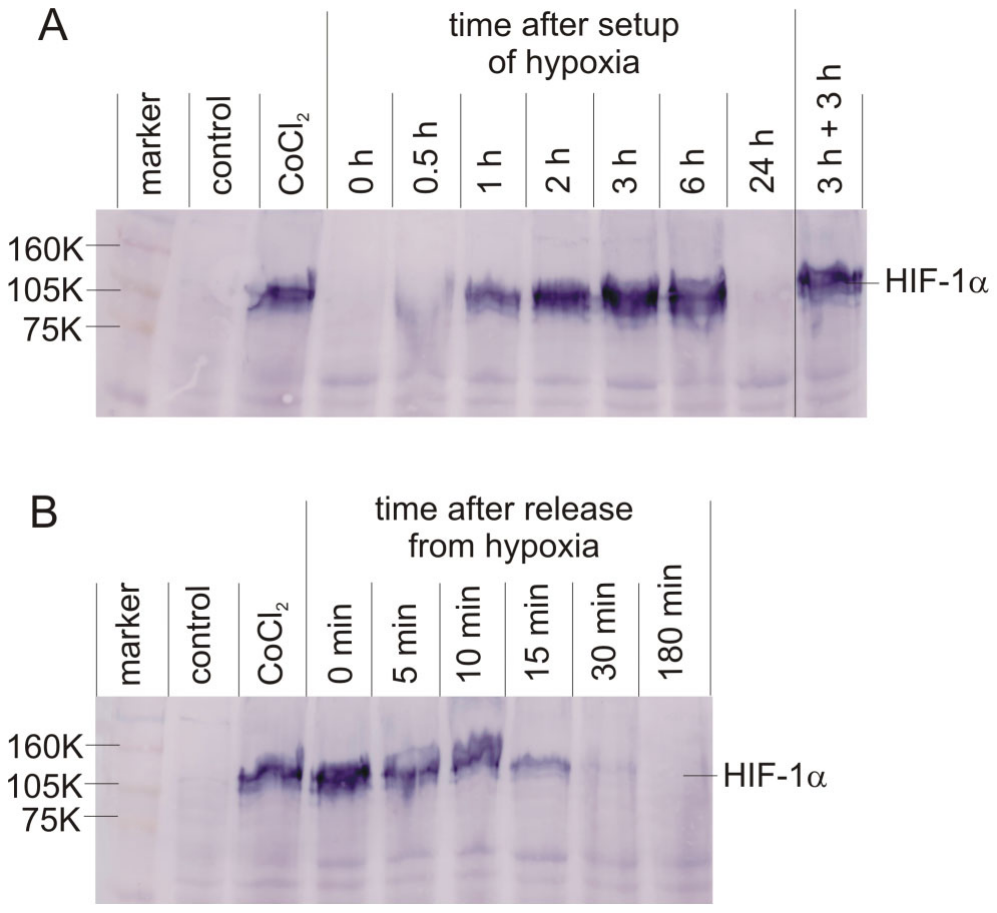
Western blot analysis of HIF-1α expression in CAL33 cells under normoxia and hypoxia. A) HIF-1α expression at different times (0–24 h) after setup of hypoxia; 3 h+3 h: incubation of cells done twice for 3 hours under hypoxia, interrupted by a 10 min phase under normoxic conditions. B) HIF-1α expression at different times (0–180 min) after release of CAL33 from hypoxia and incubation under normoxic conditions; control: normoxic cells; CoCl_2_: cells treated with CoCl_2_ used as positive control; 160 K, 105 K, 75 K molecular weight markers (RPN800, GE Healthcare). Unspecific staining of protein bands other than HIF-1α was used as sample loading control.

For that purpose CAL33 cells were lysed in M-PER buffer (Thermo Scientific, Pierce Biotechnology, Rockford, IL) in the presence of protease inhibitors (Complete Mini Protease Inhibitor, Roche, Switzerland). Cell debris was pelleted by centrifugation and protein supernatants were concentrated. Protein concentrations were determined according to the method of Bradford (BioRad, Germany). Cell lysates (47.5 µg protein per lane) were subjected to SDS-PAGE (8% acrylamide) and subsequently blotted onto a PVDF membrane. For detection of HIF-1α, the membrane was probed with primary (monoclonal anti-human HIF-1-α IgG1 from mouse, BD Biosciences) and secondary (Fc-specific goat anti-mouse IgG coupled with alkaline phosphatase, Sigma) antibodies. HIF-1-α was visualized in a colour reaction using NBT and BCIP as substrates (Fig. 2).

### Coupling of the α-emitter ^213^Bi to the anti-EGFR-MAb matuzumab

Matuzumab (Merck, Germany) was conjugated with the bifunctional chelating agent N-[2-amino-3-(p-isothiocyanatophenyl)propyl1]-trans-cyclohexane-1,2-diamine-N,N’,N’’,N’’’,N’’’’-pentaacetic acid (CHXA”-DTPA) (Macrocyclics, USA) as previously described [29]. The α-emitter ^213^Bi was eluted from an ^225^Ac/^213^Bi generator system produced by the Institute for Transuranium Elements (European Commission, JRC, Germany) [30]. Chelated anti-EGFR-mAb (100 µg) was incubated with the ^213^Bi eluate (BiI_4_^-^/BiI_5_^2-^ anionic species) in 0.4 M ammonium acetate buffer at pH 5.3 for 7 min. Unbound ^213^Bi ions were cleared from ^213^Bi-anti-EGFR-Mab immunoconjugates by size-exclusion chromatography (PD-10 columns, GE Healthcare, Germany). Purity of ^213^Bi-immunoconjugates was assayed via instant thin-layer chromatography [31]. Binding of ^213^Bi-anti-EGFR-MAb to CAL33 cells was quantified as previously described [32].

### Irradiation of CAL33 cells with ^213^Bi-anti-EGFR-MAb under normoxic and hypoxic conditions

CAL33 cells were seeded in 96-well plates (1×10^4^ cells per well) or 24-well plates (200 cells per well) for use in cell viability assays or clonogenic assays, respectively. For adjustment of cell metabolism to hypoxia, CAL33 cells were incubated in the aluminium chamber under hypoxic conditions for 3 hours before irradiation. ^213^Bi-anti-EGFR-MAb (9.25 kBq–1.48 MBq) was added within 10 min after removal of cells from the aluminium chamber. Subsequently, cells were again incubated under hypoxic conditions for 3 hours. Cells were then kept under normoxic conditions until evaluation of viability and survival. Treatment of CAL33 cells with ^213^Bi-anti-EGFR-MAb under normoxic conditions was done in an analog setting.

### Irradiation of CAL33 cells with linear accelerator photons under normoxic and hypoxic conditions

CAL33 cells (4.5×10^6^) were seeded in 25 cm^2^ culture flasks. Culture flasks were incubated in the aluminium chamber under hypoxic conditions for 3 hours. Subsequently hypoxic cells inside the chamber were irradiated from outside with photons from a linear accelerator (Varian Clinac DHX) with various doses ranging from 0.5–12 Gy (nominal photon energy 6 MeV) as shown in Fig. 1. After irradiation cells were kept inside the aluminium chamber under hypoxic conditions for further 3 hours. CAL33 cells kept under normoxic conditions were irradiated with photons also in the aluminium chamber (under normoxic conditions). After irradiation and 3 h incubation under hypoxic or normoxic conditions, cells were detached, seeded in 96-well plates (1×10^4^ cells per well) or 24-well plates (100 cells per well) and incubated under normoxic conditions until evaluation of cell viability/survival.

### Cell viability assay and clonogenic assay

For colorimetric determination of cell viability/metabolic activity, CAL33 cells were incubated with the water-soluble tetrazolium salt 1 (WST-1, Roche, Germany). The rate of WST-1 cleavage to a formazan dye by mitochondrial dehydrogenases correlates with the number of viable cells. Formation of the formazan dye was quantified with a scanning multi-well spectrophotometer (ELISA reader, BioTek, USA). 3.5 days after treatment of cells in 96-well plates, culture medium was replaced with 100 µl of fresh culture medium and 10 µl of WST-1 was added. Absorbance was measured at 440 nm 1.5 h later.

Cell survival was determined using the clonogenic assay. For that purpose cells were seeded in 24-well plates and the number of cell clones consisting of more than 50 cells was counted microscopically 4.5 days after treatment of cells.

### Statistical analysis

Statistical analysis was performed using the statistical software program R, version 2.12.2 (R Foundation for Statistical Computing, Vienna, Austria). All statistical analyses are based on at least 5 independent experiments. To compare the results of the different experiments, a t-test according to Welsh and a parameter test on *β* = 0 in a Generalized Linear Mixed Model (GLMM) were used. The t-test evaluates whether the means of cell survival and cell viability under normoxia and hypoxia differ significantly. For the comparison of controls an analysis of variance (ANOVA) was conducted additionally. The GLMM takes into account different conditions that could occur during independent experiments by inclusion of the experiment number as random effect. The exponential function of the model parameter for oxygenation can be interpreted as relative risk factor (RR), denoting exp(*β*)-/RR-fold higher survival for normoxia than for hypoxia. The level of significance was set to *α* = 0.05.

## Results

### HIF-1α expression in CAL33 cells under normoxic and hypoxic conditions

To monitor the hypoxic status of CAL33 cells, expression of HIF-1α was analysed via western blotting. HIF-1α could be detected for the first time 30 min after incubation of CAL33 cells under hypoxic conditions and reached a maximum after 3 hours (Fig. 2A). Cells were also truly hypoxic after 6 hours under hypoxia (Fig. 2A, 6 h) and after incubation done twice for 3 hours under hypoxia, interrupted by a 10 min phase under normoxic conditions (Fig. 2A, 3 h+3 h). The 6 h period and the 3 h+3 h period represent times under hypoxia of CAL33 cells irradiated with photons and incubated with ^213^Bi-anti-EGFR-MAb, respectively. After 24 hours under hypoxia, cells had died and HIF-1α could no longer be detected (Fig. 2A, 24 h).

**Figure 3:**
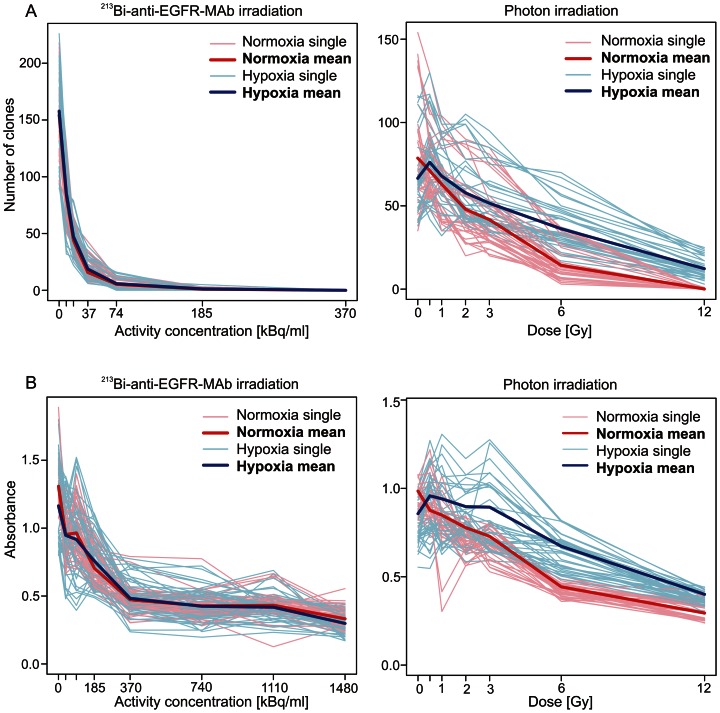
Clonogenic survival and viability of normoxic and hypoxic cells after irradiation with photons or ^213^Bi-anti-EGFR-MAb. Results of single experiments are shown as pale coloured graphs, results of means as bold coloured graphs. A) Number of clones (clonogenic assay) and B) absorbance (WST viability assay) as a function of activity concentration (^213^Bi-anti-EGFR-MAb) or photon dose, respectively.

In CAL33 cells that had been incubated under hypoxic conditions for 3 hours, release from hypoxia and cultivation under normoxic conditions resulted in a decrease of the HIF-1α signal. The HIF-1α signal could still be detected 15 min after release from hypoxia. After 30 min HIF-1α had almost disappeared (Fig. 2B).

### Survival and viability of CAL33 cells after incubation under hypoxic conditions

To evaluate the consequences of hypoxia on survival and viability, CAL33 cells were incubated under hypoxia corresponding to the treatment protocols. Survival and viability of the cells were compared with cells kept under normoxia. No significant difference with respect to cell survival (clonogenic assay) could be observed between normoxic cells and cells transiently kept under hypoxic conditions in almost all statistical evaluations. Regarding the WST assay statistical tests indicated significantly lower cell viability of hypoxic cells.

### Survival of CAL33 cells after irradiation with photons or with ^213^Bi-anti-EGFR-MAb under normoxia and hypoxia

The number of CAL33 cell clones decreased with increasing dose of photons, both under normoxic and hypoxic conditions. However, at every dose, survival of CAL33 cells incubated under hypoxic conditions was significantly higher than survival of cells incubated under normoxic conditions. After irradiation with 12 Gy the mean number of clones amounted to 0.1 (standard deviation SD = 0.3) for normoxic cells and to 12.3 (SD = 5.8) for hypoxic cells (Fig. 3A). This clearly demonstrates that hypoxic cells are less sensitive towards photon irradiation than normoxic cells.

Incubation of CAL33 cells with ^213^Bi-anti-EGFR-MAb resulted in an excellent binding of immunoconjugates (78.3%, SD = 8.0), as a prerequisite for high cytotoxicity. Corresponding to photon irradiation, the number of CAL33 cell clones decreased with increasing ^213^Bi-anti-EGFR-MAb activity concentration both for cells irradiated under hypoxic and normoxic conditions. In contrast to photon irradiation the number of cell clones did not differ significantly for cells irradiated under normoxic or hypoxic conditions at any given activity concentration. For example, at activity concentrations of 9.25 kBq/ml and 74 kBq/ml the mean number of clones accounted for 83.3 (SD = 19.8) and 5.3 (SD = 3.1) under normoxia, as well as 85.8 (SD = 23.6) and 5.8 (SD = 4.2) under hypoxia, respectively. After incubation with 370 kBq/ml ^213^Bi-anti-EGFR-MAb none of the cells survived both under normoxia and under hypoxia (mean = 0; SD = 0) (Fig. 3A). This indicates that targeted treatment with the α-emitter ^213^Bi eradicates normoxic and hypoxic cells with identical efficiency.

### Viability of CAL33 cells after irradiation with photons or with ^213^Bi-anti-EGFR-MAb under normoxia and hypoxia

Viability of CAL33 cells was determined using the WST assay. Cell viability decreased with increasing doses of photon irradiation. CAL33 cells treated under hypoxia showed superior viability compared to cells irradiated under normoxia (Fig. 3B). For example, absorbance after irradiation with 3, 6 and 12 Gy was 0.73 (SD = 0.10), 0.44 (SD = 0.06) and 0.29 (SD = 0.05) for normoxic cells and 0.89 (SD = 0.19), 0.67 (SD = 0.10) and 0.40 (SD = 0.06) for hypoxic cells, respectively. This is in accordance with the results of the clonogenic assay.

After incubation with increasing activity concentrations of ^213^Bi-anti-EGFR-MAb (37 to 370 kBq/ml), cell viability progressively decreased. At an activity concentration of 370 kBq/ml nearly all cells had been eradicated. Only due to remaining cell-debris the absorbance did not decrease to zero but remained at 0.3 to 0.4 for lethal activity concentrations ranging from 370 kBq/ml to 1.48 MBq/ml of ^213^Bi-anti-EGFR-MAb. Cell viability was affected identically in hypoxic and normoxic cells (Fig. 3B). Again, these results are in contrast to the effects of photon irradiation and are in accordance with those of the clonogenic assay after ^213^Bi-anti-EGFR-MAb irradiation.

### Oxygen enhancement ratios (OER) for survival and viability of CAL33 cells

The OER is defined as the ratio of radiation doses required to produce the same biological effect in cells kept under hypoxia and normoxia. As deduced from the data presented in Fig. 3, the OER for cell survival varied between 1.5 and 1.9 (mean 1.7; SD = 0.1). Accordingly, the OER for cell viability varied between 1.6 and 2.3 (mean 1.8; SD = 0.2). In contrast, after irradiation with ^213^Bi-anti-EGFR-MAb under hypoxic and normoxic conditions both survival and viability did not differ significantly as expressed by OER values of 1, respectively (data not shown).

### Statistical evaluation of irradiation effects under normoxic and hypoxic conditions with regard to overall survival and viability of CAL33 cells

**Figure 4:**
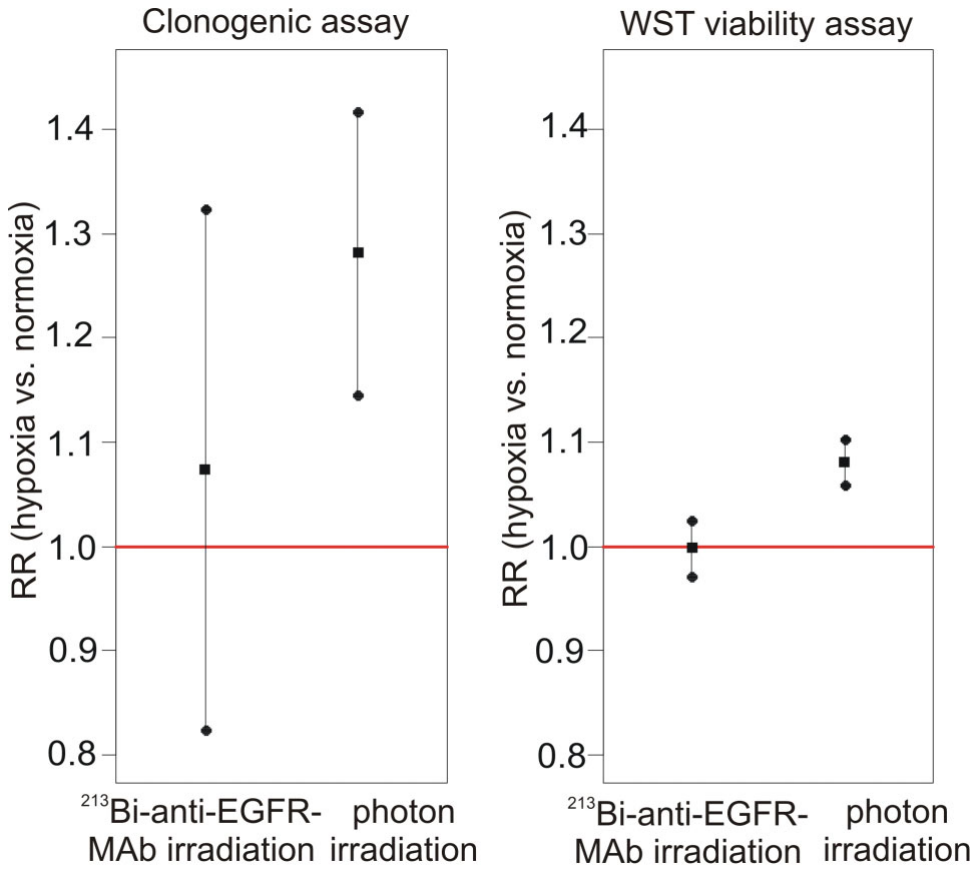
Relative risk for cell survival/viability after irradiation with photons/^213^Bi-anti-EGFR-MAb under normoxia/hypoxia. Relative risk (RR) (▪) denotes RR-fold higher survival of CAL33 cells irradiated under hypoxia compared to normoxia. RR was calculated according to the Generalized Linear Mixed Model (GLMM). Overall cell survival/viability is significantly (RR-fold) higher under hypoxia, if the 95%- confidence interval (•) doesn't include the value 1 (red line).

With regard to overall survival of CAL33 cells after photon irradiation both statistical methods revealed that the number of clones after irradiation under hypoxia was significantly higher compared to irradiation under normoxia (GLMM: p = 0.000; t-test: p = 0.000; data not shown). According to the GLMM the relative risk (RR) was 1.28 (Fig. 4). In contrast, overall survival of CAL33 cells after irradiation with ^213^Bi-anti-EGFR-MAb under hypoxic and normoxic conditions did not differ significantly in the GLMM with a RR of 1.07 (p = 0.547) (Fig. 4). Evaluation of the results with the t-test confirmed that there is no difference between hypoxia and normoxia (p = 0.547; data not shown).

Statistical analyses of the results of the WST viability assay were in accordance with those of the clonogenic assay. Overall cell viability was significantly higher after photon irradiation under hypoxic conditions compared to normoxic conditions with a RR of 1.08 (p = 0.000), according to the GLMM and confirmed by the t-test (p = 0.000; data not shown) (Fig. 4). Moreover, irradiation of CAL33 cells with ^213^Bi-anti-EGFR-MAb under hypoxic and normoxic conditions did not result in a statistically significant difference concerning overall cell viability, both according to the GLMM (RR = 1.00; p = 0.950) and to the t-test (p = 0.933; data not shown) (Fig. 4).

### Statistical evaluation of irradiation effects under normoxic and hypoxic conditions with regard to survival and viability of CAL33 cells at different doses/activity concentrations

**Figure 5:**
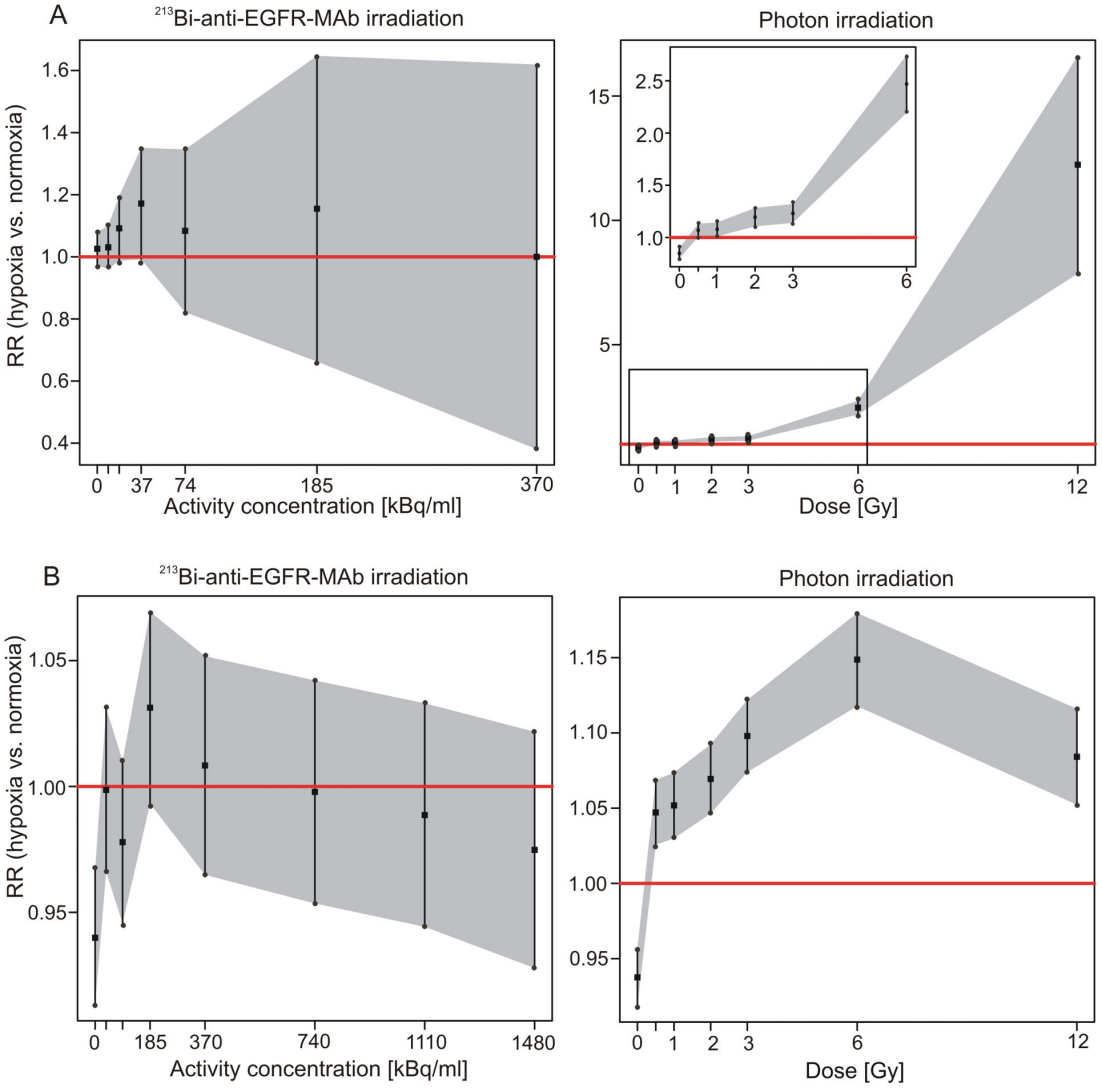
Relative risk for cell survival (A)/viability (B) at different photon doses/^213^Bi-anti-EGFR-MAb activity concentrations under normoxia/hypoxia. Relative risk (RR) (▪) denotes RR-fold higher survival of CAL33 cells irradiated under hypoxia compared to normoxia. RR was calculated according to the Generalized Linear Mixed Model (GLMM). Overall cell survival/viability is significantly (RR-fold) higher under hypoxia, if the 95%- confidence interval (•) doesn't include the value 1 (red line).

The GLMM data from the clonogenic assays revealed that survival of CAL33 cells after photon irradiation under hypoxic conditions was significantly higher than under normoxic conditions at each dose analysed. Moreover, the RR increased with increasing doses, i.e. from 1.07 at 0.5 Gy, to 12.24 at 12 Gy. After irradiation with ^213^Bi-anti-EGFR-MAb, cell survival was not significantly different under hypoxia or normoxia at any given activity concentration (Fig. 5A).

The results obtained from the clonogenic assay could be confirmed with the GLMM data from the WST cell viability assay. After photon irradiation under hypoxic conditions metabolic activity of CAL33 cells was significantly higher than after irradiation under normoxic conditions at each dose analysed. The maximum RR was observed at 6 Gy (1.15). At 12 Gy the RR was 1.08. In contrast, after irradiation of cells with ^213^Bi-anti-EGFR-MAb, cell viability was not significantly different at any activity concentration investigated in terms of irradiation effects under hypoxia and normoxia (Fig. 5B).

## Discussion

Hypoxic areas within solid tumors are still a major problem in cancer treatment. There are obvious connections between hypoxia and (i) resistance towards radiation therapy and chemotherapy, (ii) acquirement of invasive and metastatic properties and (iii) a poor clinical prognosis [33]. Hypoxia triggers radioresistance by two different ways. As cellular oxygen concentration decreases, i.e. as hypoxia increases, radiosensitivity of tumor cells decreases accordingly, a phenomenon that was termed the oxygen effect [18]. This is obviously due to a reduced formation of radicals finally causing DNA damage, being strictly dependent on oxygen supply. Furthermore, the duration of hypoxia influences radiosensitivity of tumor cells [34]. Thus the second way how hypoxia triggers radioresistance is due to physiological changes of hypoxic tumor cells. As a consequence of reduced p[O_2_] levels, hypoxia inducible factors (HIFs) modulate cellular processes via regulation of transcription, which finally affect radioresistance towards photon irradiation [6], [7], [11]. This implicates that hypoxia induced radioresistance is dependent on adjustment of cell metabolism to hypoxic conditions. To accommodate this fact, CAL33 cells were incubated for 3 hours under hypoxia before irradiation. As we have demonstrated, HIF-1α appeared 30 min after incubation of CAL33 cells under hypoxia and reached maximum expression after 3 h. Moreover, HIF-1α could be detected at any time of irradiation of CAL33 cells under hypoxia, thus ensuring the hypoxic status (Fig. 2). In addition, oxygen pressure in the culture medium was <10 mmHg 9 min after onset of hypoxic conditions. Assuming that reduced radiosensitivity due to hypoxia appears at p[O_2_] <10 mmHg [4], [5], during a 180 min incubation with ^213^Bi-anti-EGFR-MAb, ^213^Bi acts on truly hypoxic cells for a period of 171 min (180 min minus 9 min). This corresponds to 80.7% of the applied ^213^Bi activity (t½ = 46 min). Again this demonstrates that the protocols used for irradiation of CAL33 cells under hypoxic conditions were adequate and that cells were truly hypoxic at the time of irradiation.

The results of statistical analysis revealed that both survival and viability of hypoxic CAL33 cells after photon irradiation were significantly higher compared to normoxic cells (Fig. 4). For cell survival, the oxygen enhancement ratio (OER) varied between 1.5 and 1.9 (mean 1.7; SD = 0.1). Accordingly, the OER for cell viability varied between 1.6 and 2.3 (mean 1.8; SD = 0.2) (data not shown). These results are in accordance with previously published OER values [18]. In contrast, both survival and viability after irradiation with ^213^Bi-anti-EGFR-MAb under hypoxic and normoxic conditions did not differ significantly (Fig. 4). The OER was 1, respectively, confirming that there is no difference with regard to cytotoxicity (data not shown). This again demonstrates efficacy of alpha-emitters in terms of eradication of hypoxic cells.

The impact of incubation of CAL33 cells under hypoxic conditions turned out to be negligible in terms of survival. Incubation of cells under hypoxia – without irradiation – did not decrease clonogenic survival, measured 4.5 days after hypoxia, compared to cells that were grown under normoxia throughout. However, analysis of cell viability 3.5 days after hypoxia showed slightly higher viability of normoxic cells, indicating that hypoxia might have caused enduring changes in cell metabolism. Also the RR for cell viability was less than the RR for cell survival (Figs. 4, 5). The different results can be due to the different times of measurement for cell viability and cell survival or most likely, due to the different end-points that are determined by the different assays. Nevertheless, these results are not contradictory to the oxygen independent eradication of CAL33 by ^213^Bi-anti-EGFR-MAb.

The RR for both cell survival and cell viability increased with increasing doses of photon irradiation. For cell survival the RR increased from 1.07 at 0.5 Gy, to 12.24 at 12 Gy (Fig. 5A). This indicates that the effect of radioresistance on survival of hypoxic tumor cells increases with increasing doses of photon irradiation. This is assumed to be due to the mechanism of DNA-damage induction after photon irradiation. In the presence of oxygen (normoxia) radiation induced formation of radicals that finally cause DNA-damage increases with increasing doses. In the absence of oxygen, formation of radicals is limited even at high irradiation doses. The maximum RR for cell viability was observed already at 6 Gy (RR = 1.15). At 12 Gy the RR was 1.08. This is due to the converging graphs of Fig. 3B. For normoxia the minimum of absorbance was already reached at 6 Gy. Thus, the difference between normoxia and hypoxia gradually decreased between 6 and 12 Gy.

In contrast, after irradiation with ^213^Bi-anti-EGFR-MAb cell survival and viability were not significantly different under hypoxia and normoxia at any activity concentration investigated (Fig. 5). This demonstrates that the effect of alpha-particle radiation is independent of free radical formation. Densely ionizing, high LET alpha-particle radiation directly produces DNA double-strand breaks with high efficiency, thus causing multiple breaks within single chromosomes independent of cellular oxygenation. Therefore, alpha-particles emitted by ^213^Bi immunoconjugates can overcome the oxygen enhancement effect as observed for photon irradiation in CAL33 cells.

This characteristic of the alpha-emitter ^213^Bi could possibly be exploited in cancer treatment. Hypoxic regions within solid tumors are associated with increased resistance to radiation and chemotherapy and therefore are considered as a negative prognostic factor in various types of cancer [4], [35]. Therefore, new therapeutic options specifically addressing treatment of hypoxic tumor areas are needed.

Cytotoxicity of irradiation of hypoxic cells with alpha-emitters such as ^210^Po and ^238^Pu from an external source in comparison to low LET emitters has been evaluated in previous studies, for example by Jenner et al. [36] and by the group of Barendsen [37]. It could be demonstrated that the OER decreases with increasing LET of the applied radiation. The results of these studies suggested that application of high LET alpha-particles seems promising in therapy of hypoxic tumors. However, the external irradiation studies suffer from methodical inadequateness. For external irradiation with alpha-emitters under hypoxic conditions cells had to be seeded on ultrathin foils. This resulted in (i) oxygen influx [38], (ii) inhomogeneous dose distribution and (iii) an insufficient irradiation of the whole cell [39]. Consequently OER findings varied considerably as shown in the work of Wenzl and Wilkens [18]. Moreover, these studies did not consider ways of application of alpha-emitters to tumors, neglecting that external irradiation of tumors with alpha-emitters is not applicable.

EGRF is a promising receptor in targeted therapy because it is overexpressed in a variety of tumors such as bladder cancer [40] as well as head and neck cancer [41]. In fact, application of the anti-EGFR antibody cetuximab turned out beneficial in treatment of patients suffering from colorectal cancers and HNSCC [41]. However, because resistances towards anti-EGFR therapy were shown to develop in tumors, particularly in those with high EGFR expression [26], coupling of cytotoxic compounds to the antibodies is a promising strategy to overcome resistances. Therefore, coupling of compounds that efficiently eradicate hypoxic tumor cells, such as α-particle emitting radionuclides, seems a promising strategy. As we have demonstrated in vitro, the α-emitter ^213^Bi coupled to the anti-EGFR antibody matuzumab efficiently kills hypoxic tumor cells. However, targeting of hypoxic tissue in vivo with ^213^Bi-anti-EGFR antibody conjugates could be impaired due to the poorly vascularized microenvironment of hypoxic tumors [33]. Targeting of hypoxic tissues in vivo could be improved by application of smaller carrier molecules that show a better tissue penetration than antibodies. This could include Fab fragments of anti-EGFR antibodies and single chain antibodies (scFv) efficiently targeting EGFR.

Moreover, targeting of hypoxic tumor cells with the α-emitter ^213^Bi could possibly be achieved independently of EGFR targeting. For that purpose drugs that preferentially target hypoxic tumor cells should be modified in such a way that they can be labeled with the α-emitter ^213^Bi. Among the dithiosemicarbazone compounds the hypoxia-selective agent diacetyl-bis(N(4)-methylthiosemicarbazone) labeled with the high LET Auger-emitter ^64^Cu (^64^Cu-ATSM) was already shown to increase DNA-damage and cytotoxicity in hypoxic cells [19]. Furthermore, labeling of misonidazole (MISO) and azomycinarabinofuranoside (AZA) of the nitroimidazole family of radiotracers with α-emitters via appropriate chelating agents could be a promising therapeutic strategy. Both MISO and AZA labeled with the PET tracer ^18^F (F-MISO; FAZA) were successfully evaluated in clinical studies in terms of detection of hypoxic tumors [42]. In summary, targeting of hypoxic tumor cells with α-particle emitting radionuclides is a promising strategy to overcome resistance towards photon irradiation and to improve efficacy of treatment of hypoxic tumors.
